# How to improve uptake and adherence to dietary trials in gestational diabetes amongst South Asian women: insights from patient and public involvement and engagement

**DOI:** 10.3310/nihropenres.14303.1

**Published:** 2026-06-29

**Authors:** Elizabeth Dapre, Michelle Harvie, Basil Issa, Brian McMillan, Helen Morley

**Affiliations:** 1 Centre for Primary Care and Health Services Research, The University of Manchester Centre for Primary Care, Manchester, England, M13 9PL, UK; 2 Department of Nutrition and Dietetics, Manchester University NHS Foundation Trust, Manchester, England, M13 9LW, UK; 3 Division of Cancer Sciences, The University of Manchester Division of Cancer Sciences, Manchester, England, M13 9NT, UK; 4 Diabetes, Endocrinology and Metabolic Services, Manchester University NHS Foundation Trust, Manchester, England, M13 9WL, UK; 5 Division of Psychology and Mental Health, The University of Manchester Division of Psychology and Mental Health, Manchester, England, M13 9PL, UK

**Keywords:** patient and public involvement and engagement (PPIE), South Asian women, gestational diabetes, diet, trial design

## Abstract

**Background:**

Gestational diabetes mellitus (GDM) is associated with adverse maternal and neonatal outcomes and affects approximately 10–20% of pregnancies in the UK. The prevalence of GDM among South Asian women is around twice that of White British women, and they are also twice as likely to develop type 2 diabetes following diagnosis. Although multiple dietary interventions have been explored to improve glycaemic control in GDM, no single dietary approach has proven consistently effective in improving health outcomes.

We conducted a patient and public involvement and engagement (PPIE) activity with South Asian women to inform a trial evaluating the acceptability, feasibility, and safety of an intermittent low-energy diet (ILED) in GDM. PPIE input was particularly valuable in addressing lower-than-anticipated recruitment and retention, especially among South Asian women, and provided insights into sociocultural barriers and facilitators to improve trial design and participation.

**Methods:**

Six South Asian women with lived experience of overweight or obesity in pregnancy, either personally affected by gestational diabetes or with close family experience, participated in one-to-one discussions lasting up to 60 minutes to provide feedback on trial procedures, methods, and design. Based on these insights, a follow-up discussion group was conducted with eleven South Asian women who had been pregnant in the last five years, were currently pregnant, or planning pregnancy, and who had experience of overweight/obesity or GDM. Key priorities were identified and translated into practical action points to inform ongoing research.

**Conclusions:**

Three key priorities emerged as a potential means of enhancing engagement with South Asian women: addressing vulnerability (including trust and loss of control), improving accessibility, and reducing mental load (including competing priorities, emotional wellbeing, and family influence).

## Plain Language summary

South Asian women are twice as likely to develop gestational diabetes compared to White British women but remain underrepresented in clinical trials. Recruitment challenges in a recent intermittent diet trial prompted discussions with South Asian women to improve the inclusivity of the research design.

Seventeen women (‘contributors’) from Greater Manchester, with personal or family experience of gestational diabetes, reviewed study materials and provided feedback on improving participation and trial design.

Key barriers identified included lack of trust in researchers and perceived “research fatigue,” where participants felt repeatedly asked to take part in research without seeing meaningful improvements in healthcare.

Dietary adherence during pregnancy was also considered challenging due to cravings, household responsibilities, and caregiving roles. Contributors recommended family-based meal planning, pictorial guides for healthier alternatives, and financial support for childcare to facilitate attendance at trial appointments. Flexible face-to-face and online appointment options were positively received.

Emotional wellbeing was also highlighted as a key factor influencing adherence, with suggestions for support groups to help participants manage stress and avoid using food for comfort.

These discussions informed recommendations for more inclusive research design, which are shared to support future studies.

## Introduction

Gestational diabetes mellitus (GDM) affects approximately 10–20% of pregnancies in the UK. ^1^ The prevalence of GDM amongst South Asian women is around twice that seen in White British women; the Born in Bradford study found 13% of pregnancies affected compared to 6% in White women when using NICE criteria, with rates of up to 24% seen in South Asian women when using ethnic-specific criteria. ^2^ GDM is associated with both maternal and neonatal adverse health outcomes, such as emergency caesarean section, preterm delivery, large for gestational age babies, admission to neonatal intensive care units, and childhood obesity. ^3^ Half of all women with GDM develop type 2 diabetes within five years, and South Asian women are twice as likely to be diagnosed than White women. ^4^ As highlighted in the Mothers and Babies: Reducing Risk through Audits and Confidential Enquiries (MBRRACE) report, overall maternal and neonatal outcomes remain poorer in South Asian women compared with White women, with GDM care noted as a particular area for improvement. ^5^ Whilst GDM disproportionately affects women of South Asian origin, they remain underrepresented in clinical trials. ^6^ It is imperative, therefore, that we seek to understand better ways to engage with South Asian women to work collaboratively to improve outcomes.

Dietary management alongside physical activity is important in the management of gestational diabetes. ^7^ Many diets have been studied to improve blood sugar control in gestational diabetes, however no single diet has been found to be effective for improving health outcomes for women and babies. ^8^ Patient and public involvement and engagement (PPIE) work was important at all stages of our recent trial testing the feasibility of an intermittent energy restricted diet as an intervention for GDM. ^9^ During a period of slow recruitment noted particularly amongst South Asian women, we invited women to participate in further PPIE work to gain deeper insight into how to maximise engagement and help improve the design and development of our current and future research activities.

## Methods

Patient and public involvement: this report is based on the findings of a patient and public involvement activity which was carried out to support uptake to a dietary intervention trial in gestational diabetes. Findings were used to refine research design and have been disseminated locally and nationally to support inclusive research practices.

South Asian women with lived experience of overweight or obesity in pregnancy, who had either been personally affected by gestational diabetes or who had a close family member who was, and who had been recommended to modify their diet in pregnancy, were invited to share their insights regarding our study during either a face-to-face workshop or a 45–60 minute individual online or face-to-face discussion in Greater Manchester in April 2024. Requests for input from public contributors were sent out through various channels including the antenatal clinic at Wythenshawe Hospital, local Facebook community groups, word of mouth, and third sector organisations including British Muslim Heritage Centre, Let’s Talk Rochdale and the Women’s CHAI Project. Women were asked to contact the researcher directly via e-mail or phone. We intended to hold two workshops; one with women with a history of GDM and another with partners of women with a history of GDM. Despite multiple attempts and approaches to gain interest and attendance at these workshops there was minimal response. One-to-one discussions were therefore offered, and six women responded to this invitation and preferred an online Microsoft Teams call which was arranged at a time convenient to them. All women who responded were sent information about what the PPIE activity would involve, along with the participant information leaflet (PIL) for the trial itself (Appendix 1) for review prior to the discussion to enable them to understand the trial and offer feedback on its design. Upon commencing the call, and prior to starting the discussion, all women were given the opportunity to ask questions and were given a brief reminder of the existing study design and that the purpose of the discussion was to help to improve the research design. It was reiterated that their participation was entirely voluntary, and that they were free to stop the call at any time without consequence. Only following their verbal agreement to proceed did the discussion formally begin. Personal data was not collected as this was a PPIE activity, however all women voluntarily offered their country of ethnic origin and personal experience of GDM which helped to ensure relevance to our trial population. All names were then replaced with an identification number to maintain anonymity.

A further discussion group was subsequently arranged in partnership with the Women’s CHAI Project with a different group of participants. The Women’s CHAI Project is a local charity which exists to create a safe and inclusive space for women and mothers. Eleven South Asian women who had been pregnant in the last five years, were currently pregnant, or who were planning pregnancy, and with experience of overweight/obesity or GDM, were invited to a 90-minute discussion group to consider our trial design and discuss ways to improve engagement in our research. It was held in a private, neutral location of the charity’s choosing. Contributors were provided with the PIL and written consent was obtained with no personal data beyond consent collected. With participants’ prior written agreement the discussion was audio recorded using Microsoft Teams enabling auto-transcription to allow the researcher to focus on the discussion without note taking. Transcripts were reviewed for familiarisation and deleted within one week; they contained no personal identifiers.

All discussions were in English with the same female researcher who was a White British academic GP trainee, and who introduced all discussions by confirming the purpose was to help improve and develop current and future research design. A female community peer who worked for the Women’s CHAI Project translated into Urdu where necessary during the discussion group. All women were offered £25 per hour for their time in line with NIHR rates for PPIE reimbursement.

An open questioning technique was used, employing the use of prompts if needed.

Topics discussed included:

- How women may feel taking part in the dietary research during pregnancy, including what might enable them to take part and what could prevent participation
- How a diagnosis of gestational diabetes may impact women’s decision to take part in the research
- How likely women would be to follow the intermittent low-energy diet during pregnancy, and what could make the intervention easier for women to follow
- Their views on the time of GDM diagnosis as point of recruitment to a dietary intervention
- Whether any sociocultural barriers and/or facilitators could be addressed to improve engagement with our dietary trial

Key points from the PPI activities were reviewed and synthesised to identify priority areas for practical action to improve research design.

In accordance with Health Research Authority and NIHR guidelines for PPIE, ethical approval was not required. ^10, 11^ However, the work was conducted within the ethical framework of the primary clinical trial, which included specific provisions for PPIE activity. ^9^ Ethical approval was granted from the Cambridge East Research Ethics Committee (22/EE/0119).

Six women agreed to take part in one-to-one discussions and eleven women in the discussion group and are hereon referred to as contributors. Amongst the six contibutors interviewed, five were from Pakistan and one was from Malaysia, and all identified themselves as Muslim. Eleven women attended the discussion group and all identified as South Asian. All contributors had experienced overweight, obesity, and/or had personal or family experience of gestational diabetes.

One-to-one discussions lasted between 40–60 minutes and the discussion group lasted 90 minutes.

Three broad priority areas were identified, with each encompassing up to 2–3 specific areas of focus.

### Priority 1: Vulnerability

Contributors described a sense of vulnerability which could be exacerbated by the pregnant state, which was felt to contribute to a potential fear and reluctance to take part in the research. This included both systemic vulnerability as a minoritised group and a personal loss of control throughout pregnancy, particularly when complicated by health complexities such as GDM.

### Focus area 1.1: Trust

Contributors described an underlying mistrust of both healthcare professionals and researchers based in historical abuse and discrimination of minoritised groups. One contributor described hearing about illegal activities in research in the news and recognised that many women may be put off taking part in research because of a fear of how personal information or blood samples may be used. Many of these stories were said to be passed down from previous generations who had either faced discriminatory practices or knew of someone who had been affected by unethical research activity. There was a general sense of unease about trusting those who promoted research and anxiety amongst some about how taking part may be perceived within their community. It was suggested that leaflets which explained the importance of taking part in research to the wider family could be helpful in promoting uptake.

Clear and concise information was suggested to encourage women to take part, particularly that which explained clearly how personal information would be used. It was noted that there must be a balance between adequate and transparent information provision and simplicity, as too much information can be overwhelming. Contributors suggested flexibility in information provision, with in-person explanations viewed positively.

It was suggested that word of mouth amongst the South Asian community would make women feel safer about taking part, particularly if the message was coming from a trusted voice within the community. There were varied opinions about who would be classed as a ‘trusted voice’; some viewed midwives as the best source of information and others suggested that the message would be better received from someone from the same ethnic background, language and culture. This was relevant as the main researcher approaching women in clinic for our diet study was White British, which could have reduced engagement. Seeing the same person throughout the pregnancy and/or research activity was deemed important to developing trusted relationships and maximise engagement.

Contributors suggested that women may not understand why research amongst those with gestational diabetes is important; without this understanding they may not believe that their participation is necessary or valued thereby further reducing engagement. A more overt message as to ‘why me, why now’ may increase uptake from marginalised groups, particularly as contributors suggested many women feel a sense of purpose and duty in helping their community.

### Focus area 1.2: Loss of control

Pregnancy was viewed as a time during which women may feel a loss of control, with an often unexpected diagnosis of GDM intensifying this. It was suggested that the research could be viewed as adding extra rules and restrictions during a period of already heightened stress which could be off-putting.

Dietary advice in GDM was perceived as restrictive, and taking part in research was viewed as an extra burden that women may not want. Contributors talked about rules coming from both healthcare professionals and family members, with many women referring to intergenerational and cultural beliefs around diet and physical activity during pregnancy which are likely to impact pregnant women’s health behaviours. Many contributors did recognise that taking part in research may be supportive and helpful, with additional monitoring and appointments throughout pregnancy likely to be perceived positively.

Food cravings and reduced energy levels were reported frequently amongst the contributors as powerful factors likely to limit women’s ability to control their diet during pregnancy. Food cravings amongst South Asian women were said to be perceived as the body being deficient, thus creating a perceived need to be fulfilled even if the desired foods are unhealthy. Fatigue may also reduce motivation to adhere to healthier diet plans, with sugar viewed as a quick energy source. This sense of restriction coupled with the strong physical desire for particular foods may lead to denial and poor dietary adherence.

Contributors suggested that clear tables of healthier alternatives which could satisfy cravings would be very useful and likely to improve adherence to dietary interventions.

### Priority 2: Accessibility

Accessibility to taking part was identified as a key opportunity for increasing uptake and engagement.

Childcare responsibilities were commonly cited as a barrier to taking part in the research, often reported as a barrier to being able to participate fully in routine healthcare and research appointments. It was recognised that in many South Asian households the burden of childcare falls on the woman and as such the timing and delivery of appointments is a critical factor in participation in, and adherence to, research activity, as is ensuring the proposed intervention is simple and easy to follow. Flexibility of provision of information was suggested, with written information available to supplement discussions where possible.

The study involved face-to-face clinic research appointments and remote phone/video phone dietary advice from an allocated research dietitian. This hybrid approach was viewed as beneficial, with face-to-face appointments viewed necessary to help build trusted relationships with the research team, and telephone or online appointments helpful where flexibility was required. Appointment timing during school hours was preferable and considering school holiday dates in research design was deemed likely to improve adherence and reduce attrition. Provision of childcare or reimbursement for childcare was viewed positively amongst the contributory group as a facilitator to engagement.

Information provision was raised by many contributors as a problem for three reasons:

1. Language barriers were viewed amongst most contributors as needing mitigation. For women who speak English as a second language pregnancy is already likely to present a difficult time, particularly amongst those new to the UK and facing a new healthcare system. GDM may add another layer of complexity and stress, therefore taking part in research may be viewed as an added and unnecessary burden. To maximise uptake and participation amongst such groups improvements to streamlined access and inclusivity was advised, be that through readily accessible translated materials or interpreters. Pictorial representation of meals was suggested which would be more easily accessible.
2. The amount of information available to women during pregnancy, particularly for those who face perinatal complications, can be overwhelming. Although technology and internet accessibility was viewed positively, particularly in the context of mobile applications such as the Nutritics app recommended to track dietary intake by the research team, contributors raised concerns that patients may find it difficult to assess the legitimacy of other online information. Healthcare approved applications and websites were therefore preferred, particularly if they were available in multiple languages.
3. Contributors raised a general lack of understanding about the health risks of living with overweight and obesity, and the importance of following dietary recommendations in pregnancy. They suggested that many women are encouraged by friends and family to eat more during pregnancy to support the baby’s development, contradicting the trial’s intervention. Increasing awareness of the importance of health behaviours in pregnancy to reduce maternal, neonatal and long-term adverse health outcomes was believed likely to increase uptake and adherence to dietary and physical activity interventions.

### Priority 3: Mental load

Contributors described that women feel an overriding responsibility to others, both to their unborn baby and the wider family, which often means that women’s personal needs are not prioritised. This was felt likely to have reduced willingness to take part in the research.

### Focus area 3.1: Competing priorities

There was a general acceptance amongst all contributors that the South Asian woman’s role is childcare, household responsibilities, and looking after others. This was deemed a greater priority than women’s personal needs, including their own health needs. This was felt to leave little time for participation in additional research activity.

Contributors suggested that in South Asian culture there is often an expectation that meals are cooked from scratch, usually tailored to the wishes of other family members. Providing dietary alternatives which meant one meal could be cooked for the whole family with minimal adaptations to make them suitable for the woman with GDM was advised to increase adherence. Food-based healthy eating plans with self-selected food choices were viewed favourably. Calorie controlled ready meals or total diet replacement formula shakes were not deemed an acceptable alternative unless they were used as a last resort for women who were too exhausted to cook or unable to follow dietary plans. Daily or weekly meal plans which reduced the mental burden of meal preparation were suggested as a helpful means of improving uptake and adherence to the recommended diets.

### Focus area 3.2: Emotional wellbeing

Contributors frequently raised the importance of emotional wellbeing amongst pregnant women. They noted that if women’s mental health is not supported their motivation for self-care is likely to be low, and consequently it is unlikely that they would take on the added burden of the research activity.

Women only support groups were viewed positively, as sharing experiences and meeting other women was viewed as emotionally empowering and motivating.

There was a strong recognition of the link between emotional state and food intake, and the need to support emotional wellbeing to reduce the likelihood of turning to food as comfort. Ensuring that wellbeing is supported was suggested as a key factor in improving adherence to dietary interventions.

Contributors also raised a sense of research fatigue amongst many South Asian people. They suggested that despite acknowledging the importance of participation in research, many were frustrated that research activities often did not translate to tangible changes in healthcare services for marginalised communities, and was likely to result in progressive disengagement from research activity.

### Focus area 3.3: Family influence

The significance of how friends and family may perceive antenatal health behaviours was raised as a concern, with some contributors recognising that intergenerational pregnancy beliefs may be outdated yet may be difficult to challenge due to traditionally accepted familial hierarchical structures. Contributors described a cultural acceptance that pregnant women should ‘eat for two’, and that weight gain is viewed as a positive sign in pregnancy regardless of a woman’s preconception weight. Contributors noted that many older generations may view dieting and physical activity in pregnancy negatively, therefore leaving women in a predicament between satisfying sociocultural norms and family pressures with their own health beliefs and needs. This was felt likely to result in reduced uptake to, and withdrawal from, our dietary intervention. Contributors recognised that diabetes may be viewed as an accepted part of life amongst the South Asian community, with many viewing it as an inevitability rather than something which could be affected by health behaviour change.

A ‘whole family approach’ was seen as very attractive with contributors commenting on the potential intergenerational health benefits as a motivating factor for engagement. Many suggested that leaflets for friends and family could improve family support and understanding thereby increasing health behaviour motivation and improving adherence to the intervention.

## Discussion

This patient and public involvement work aligns with existing literature highlighting mistrust of healthcare professionals and researchers amongst marginalised communities, compounded by poor communication (including language barriers and inadequate cultural competence), and the role of sociocultural influence in decision making. ^12– 14^ These challenges may be particularly pronounced for South Asian women who not only face racial discrimination but also the intersecting impact of gender bias in both healthcare and broader society. Research practices that offer practical support and reduce participant demands are more likely to improve engagement.

Although pregnancy is perceived as a time which could increase motivation for behaviour change, the advisory group emphasised that the mental load of pregnancy can be substantial and act as a barrier to change. ^15^ The added medical complexities of GDM may negatively impact on women’s mental health, potentially reducing their willingness to participate in research activities. Given the perceived emotional and psychological connections with food intake, particularly during pregnancy, adequate emotional support throughout the perinatal period is arguably critical to the success of dietary interventions. This is particularly important amongst South Asian women who often also face specific sociocultural barriers to accessing mental health support. ^16^ Our future research activities are considering how to integrate emotional support, for example through peer champions or community groups.

The value of the ‘trusted voice’ is critical in promoting research engagement amongst marginalised communities. This was reflected in the PPIE activity itself whereby collaboration with a local charity facilitated more effective recruitment to the discussion group. Beyond trial design, this activity fostered a sustained partnership with the local charity, which evolved into a series of community-led educational events about GDM and diabetes. This reciprocal approach addresses the ‘trust’ barrier identified by contributors and demonstrates how PPIE can transition from information collection to active community knowledge exchange.

Recognising and addressing health inequalities and systemic racial biases in healthcare and research, alongside enhancing cultural competence amongst research teams and healthcare professionals, is fundamental to improving trust and engagement amongst South Asian women. Targeted action in this area is essential to increasing research participation and implementing sustainable changes to advance health equity. Crucially, research must prioritise applied outcomes, ensuring that findings translate into actionable change rather than remaining purely exploratory.

### Strengths and limitations

Limitations of this work include that the researcher was White British and therefore may have been seen as an ‘outsider’, limiting trust and potentially the contributors’ willingness to be completely open. The PPIE group was relatively small, meaning that other potentially relevant themes to the South Asian community may not have been identified.

Strengths included that the majority of the contributory group were South Asian women with experience of pregnancy, overweight, obesity, and/or gestational diabetes.

## Conclusion

This PPIE work outlines key priority areas for improving engagement in dietary research in GDM with South Asian women. Pregnancy is recognised as a time of vulnerability for most women, and whilst this motivates some to take part in research it can result in a lack of engagement from others. South Asian women may hold specific hierarchical family responsibilities and experience sociocultural pressures to conform to traditional beliefs and practices surrounding pregnancy. Harnessing the collaborative power of the wider family, healthcare professionals and research teams through improved communication and cultural competence offers an important opportunity for both improved perinatal care and intergenerational health behaviour change.

The results of this engagement work were used to inform the latter stages of our trial to improve recruitment and adherence, and have directly informed the development of a broader research program focused on interpregnancy weight management in women with a high risk of cardiometabolic disease, moving away from a purely clinical focus to a more holistic, culturally-embedded model of care.

This work highlights the importance of early engagement with a diverse PPIE group to ensure accessible and relevant research design and delivery for all participants.

### Recommendations for future research

Future research activities must focus on inclusivity, particularly in ensuring simplicity of language in patient materials, practical accessibility including childcare provision, and translation of materials and use of interpreters.

Pictorial representations of meals and snacks with listed benefits for mothers and babies are likely to improve adherence to dietary interventions, particularly those which offer alternatives to common cravings.

The mental health and emotional wellbeing of women must be prioritised. There is much published in the literature regarding higher rates of emotional distress and mental health problems in minoritised ethnic groups in the UK, particularly amongst the South Asian community. ^17^ This must be considered carefully and better supported in both healthcare and research design and delivery, particularly given the strong interplay between emotional wellbeing and health behaviours. ^18^

Research bodies as a whole need to work on building trusted relationships with minoritised communities to ensure voices from those with lived experience are amplified, for example through partnerships with representative third sector organisations. Research teams, ethics committees and study sponsors must work collaboratively to reduce the burden of overwhelming information for potential study participants.

Cultural differences between groups must be acknowledged and appreciated to ensure interventions are appropriate and acceptable. A ‘whole family’ approach to dietary and health behaviour interventions in the South Asian community is likely to be most successful, as are simple interventions which offer health and wellbeing benefits without increasing a woman’s mental load.

Research projects must focus on applied outcomes which deliver tangible benefits for the communities that need them most. They should also include robust dissemination strategies to ensure the resulting impact is clearly demonstrated, to avoid PPIE fatigue.

## Ethics approval

The main feasibility trial was granted ethical approval by the Cambridge East Research Ethics Committee (22/EE/0119). The PPIE activities reported in this paper were conducted to inform the trial design. In accordance with Health Research Authority (HRA) guidance, formal Research Ethics Committee approval is not required for PPIE activities. ^10, 11^

## Data Availability

Data sharing is not applicable to this article as no new datasets were created. This report describes a PPIE activity which does not constitute primary research. Insights were collected via contemporaneous notes and audio recordings were used as a memory aid prior to being discarded to protect contributors’ privacy. All insights from public contributors are summarised within the article.
