# Supplementary material for: How to improve uptake and adherence to dietary trials in gestational diabetes amongst South Asian women: insights from patient and public involvement and engagement
Source: NIHR Open Res. 2026 Sep 29;6:77. Originally published 2026 Jun 29. [Version 3] doi: 10.3310/nihropenres.14303.3 (PMC13575402; doi:10.3310/nihropenres.14303.3)
Supplement: Peer review history

# Reviewer report: Reviewer response for version 2

**DOI:** 10.3310/nihropenres.15749.r42206

**Published:** 2026-09-14

**Authors:** Anjali Shah

**Affiliations:** 1 University of Oxford, Oxford, England, UK

An excellent paper on PPIE work, with important findings for better approaches for inclusion of South Asian women in research. While the focus is gestational diabetes, I believe the findings are generalisable to including South Asian women in all aspects of research - noting the particular set of issues they face that limit participation.

 Some comments and questions:

 1. The second paragraph of the Abstract is really Methods rather than Background - and in my opinion too long. I would suggest reducing it and increasing the size of the Abstract Conclusion, which is currently not as interesting as the Plain Language Summary.

 2. Methods: The six women taking part in one-to-one discussions are described as being from Pakistan and one from Malaysia and described themselves as Muslim. Do you have this level of information on the 11 women who took part in the discussion group?

 3. Methods: it is noted that the women either had gestational diabetes or had a family member with it. Do you have information on the numbers of women in each group?

 4. Competing responsibilities such as childcare as described. Was any indication given of who would provide the childcare e.g., another family member or how would an external care provider be viewed? Is the incentive more time or financial?

 5. Family hierarchy is mentioned. Any additional information on whose views are important e.g., husband, in-laws, parents?

 6. Given the focus on the wellbeing of the baby ahead of the pregnant women from a culture perspective (e.g., to eat for 2 and to satisfy any cravings for the baby), was there anything about slightly more emphasis on the benefits to the baby if the pregnant women took part in research (e.g., to avoid the conditions affecting the baby mentioned in the first paragraph of the Background)?

 7. Cooking meals from scratch is described in Focus area 3.1. Does the trial/research studies routinely provide alternative healthier options for traditional South Asian meals?

 8. A limitation of the work is cited as the researcher being White British. I suspect there are pros and cons to the ethnicity of the researcher, and perhaps the women having choice on whom they speak with might help? They may be more comfortable speaking with someone from the same ethnicity, or they may wonder about pre-conceived ideas and speak differently as a result. Speaking with a White British women may reveal other dimensions and inhibit other dimensions. It would be interesting in future to explore what issues arise based on interviewer ethnicity.

 The majority of these comments and points are recommendations for future exploration of these issues to encourage South Asian women to participate in both PPIE work and research.

 This paper is important and needs to be indexed to promote future developments in PPIE work.

Is the work clearly and accurately presented and does it cite the current literature?

Yes

If applicable, is the statistical analysis and its interpretation appropriate?

Not applicable

Are all the source data underlying the results available to ensure full reproducibility?

No source data required

Is the study design appropriate and is the work technically sound?

Yes

Are the conclusions drawn adequately supported by the results?

Yes

Are sufficient details of methods and analysis provided to allow replication by others?

Partly

Reviewer Expertise:

Medical epidemiology and clinical trial manager. Formerly worked on the MBBRACE and UKNeS programmes of maternal research in Oxford.

I confirm that I have read this submission and believe that I have an appropriate level of expertise to confirm that it is of an acceptable scientific standard.

# Response

**Published:** 2026-09-23

**Authors:** Elizabeth Dapre

**Affiliations:** FBMH, The University of Manchester, Manchester, England, UK

*The authors thank the reviewer for their supportive and constructive comments. We have responded to each comment below.*

 1. The second paragraph of the Abstract is really Methods rather than Background - and in my opinion too long. I would suggest reducing it and increasing the size of the Abstract Conclusion, which is currently not as interesting as the Plain Language Summary. 

*Thank you for this suggestion, the abstract has been amended accordingly.*

 2. Methods: The six women taking part in one-to-one discussions are described as being from Pakistan and one from Malaysia and described themselves as Muslim. Do you have this level of information on the 11 women who took part in the discussion group?

*Thank you for this query; due to the nature of the patient and public involvement and engagement activity, this level of data was not directly requested and only captured if voluntarily offered. This has now been clarified in the Methods.*

 3. Methods: it is noted that the women either had gestational diabetes or had a family member with it. Do you have information on the numbers of women in each group?

*We thank the reviewer for this query and the opportunity to clarify our sample of participants. Because this activity was a patient and public involvement and engagement exercise, formal medical history verification and structured clinical data collection was not performed and eligibility was confirmed through self-report. However, the vast majority of participants explicitly discussed their personal experience of gestational diabetes, with only one participant reporting participation solely due to a close family member's diagnosis. This has now been clarified in the Methods.*

 4. Competing responsibilities such as childcare as described. Was any indication given of who would provide the childcare e.g., another family member or how would an external care provider be viewed? Is the incentive more time or financial?

*We thank the reviewer for this insightful query. Whilst the specific details of childcare provision were not explicitly explored, contributors' accounts offered important context. Discussions suggested that many contributors experienced isolation and a sense of not being able to rely on others for support. Several contributors explained that their decision to participate was driven by a sense of duty to others, rather than for financial reward. A lack of personal time and the challenge of balancing competing caregiving responsibilities emerged as the primary barrier to engagement, rather than financial constraints. This is an important query which warrants exploration in future research.*

 5. Family hierarchy is mentioned. Any additional information on whose views are important e.g., husband, in-laws, parents?

*We thank the reviewer for this important query. In our discussions contributors highlighted the historical influence of parents and in-laws as holding the most authority in passing down sociocultural health beliefs and expectations. However, there was a recognition that younger generations may be more likely to challenge these traditional family hierarchies, placing greater trust in their healthcare teams, with husbands frequently viewed as allies in supporting healthcare decisions. Clarifying roles within the family hierarchy and influence in healthcare decision making requires more detailed exploration in future research.*

 6. Given the focus on the wellbeing of the baby ahead of the pregnant women from a culture perspective (e.g., to eat for 2 and to satisfy any cravings for the baby), was there anything about slightly more emphasis on the benefits to the baby if the pregnant women took part in research (e.g., to avoid the conditions affecting the baby mentioned in the first paragraph of the Background)?

*Thank you for raising this; this has now been clarified within Focus area 1.2.*

 7. Cooking meals from scratch is described in Focus area 3.1. Does the trial/research studies routinely provide alternative healthier options for traditional South Asian meals?

*Thank you for highlighting this important issue. Within our MIDDAS-GDM trial, South Asian diets were integrated into the dietary intervention plans. More broadly, the authors recognise the historical reliance on Eurocentric dietary models, with limited cultural adaptability. Whilst there is increasing emphasis on culturally competent trial and healthcare system design, we recognise that systemic gaps remain across routine clinical care and research.*

 8. A limitation of the work is cited as the researcher being White British. I suspect there are pros and cons to the ethnicity of the researcher, and perhaps the women having choice on whom they speak with might help? They may be more comfortable speaking with someone from the same ethnicity, or they may wonder about pre-conceived ideas and speak differently as a result. Speaking with a White British women may reveal other dimensions and inhibit other dimensions. It would be interesting in future to explore what issues arise based on interviewer ethnicity.

*The authors thank the reviewer for this pertinent suggestion.*

 The majority of these comments and points are recommendations for future exploration of these issues to encourage South Asian women to participate in both PPIE work and research.

 This paper is important and needs to be indexed to promote future developments in PPIE work.

*We thank the reviewer for their constructive comments and agree that further dedicated research is needed in this area to ensure that future trial and healthcare design is inclusive, relevant and accessible to the communities it serves.*

# Reviewer report: Reviewer response for version 2

**DOI:** 10.3310/nihropenres.15749.r42162

**Published:** 2026-09-08

**Authors:** Eleftheria Taousani

**Affiliations:** 1 International Hellenic University, Thessaloniki, Greece

The authors have addressed properly and answered the comments made by the reviewer so that their article can get the approval to be indexed.

Is the work clearly and accurately presented and does it cite the current literature?

Partly

If applicable, is the statistical analysis and its interpretation appropriate?

Not applicable

Are all the source data underlying the results available to ensure full reproducibility?

Partly

Is the study design appropriate and is the work technically sound?

Partly

Are the conclusions drawn adequately supported by the results?

Yes

Are sufficient details of methods and analysis provided to allow replication by others?

Partly

Reviewer Expertise:

GESTATIONAL DIABETES MELLITUS

I confirm that I have read this submission and believe that I have an appropriate level of expertise to confirm that it is of an acceptable scientific standard.

# Reviewer report: Reviewer response for version 1

**DOI:** 10.3310/nihropenres.15582.r41147

**Published:** 2026-08-05

**Authors:** Eleftheria Taousani

**Affiliations:** 1 International Hellenic University, Thessaloniki, Greece

The manuscript would benefit from several additional information regarding:

1. **Ethical considerations**

 

 The authors should state how the source data underlying the results available will ensure full reproducibility.

 It would be also helpful if the authors designing a diagram with the methodology steps taken.

  

1. **Results or Key Findings**

The authors should add a Results or a KEY Findings section, where they will present the three broad priority and focus areas (page 5) they were identified.

  

1. **Discussion**

The authors in the Discussion session should provide a more comprehensive overview of the current evidence related to similar, with their own, studies compare their findings and explicitly state how their study offers new research insights.

**Comments to the Authors**

 I would like to congratulate the authors on addressing an important and clinically relevant topic. The manuscript investigates an area of growing interest for GDM and the findings may have practical implications.

 However, there are several issues that should be addressed to strengthen the scientific quality of the manuscript.

1. **Ethical considerations**

 

 The authors state that the source data underlying the results are not available, they were collected via contemporaneous notes, and audio recordings were used as a memory aid prior to being discarded to protect contributors’ privacy. Considering the above, the authors should state how the source data underlying the results available will ensure full reproducibility.

 It would be also helpful, if the authors designing a diagram with the methodology steps taken.

Is the work clearly and accurately presented and does it cite the current literature?

Partly

If applicable, is the statistical analysis and its interpretation appropriate?

Not applicable

Are all the source data underlying the results available to ensure full reproducibility?

Partly

Is the study design appropriate and is the work technically sound?

Partly

Are the conclusions drawn adequately supported by the results?

Yes

Are sufficient details of methods and analysis provided to allow replication by others?

Partly

Reviewer Expertise:

GESTATIONAL DIABETES MELLITUS

I confirm that I have read this submission and believe that I have an appropriate level of expertise to confirm that it is of an acceptable scientific standard, however I have significant reservations, as outlined above.

# Response

**Published:** 2026-08-26

**Authors:** Elizabeth Dapre

**Affiliations:** FBMH, The University of Manchester, Manchester, England, UK

We thank Reviewer 1 for their review and recommendations. Our responses to each comment can be found in detail below. 

**1. "Ethical considerations **The authors should state how the source data underlying the results available will ensure full reproducibility. It would be also helpful if the authors designing a diagram with the methodology steps taken."**

*Thank you for this comment. This manuscript reports on a Patient and Public Involvement and Engagement (PPIE) activity rather than a research study. The UK Standards for Public Involvement do not require data generated through PPIE activities to be made publicly available. This encourages frank discussions and protects contributor confidentiality. This was not a piece of qualitative research, but PPIE is closer to this than to quantitative research, and so reproducibility in the positivist sense would not be an appropriate quality criterion for this work (see Braun & Clarke, 2022). Due to the nature of the activity, informal discussions did not follow a formal methodological framework. We have clarified this in the manuscript.*

**2. "Results or Key Findings **The authors should add a Results or a KEY Findings section, where they will present the three broad priority and focus areas (page 5) they were identified." 

*We thank the reviewer for this comment, a title has been added accordingly.*

**3. "Discussion **The authors in the Discussion session should provide a more comprehensive overview of the current evidence related to similar, with their own, studies compare their findings and explicitly state how their study offers new research insights." 

**

*We thank the reviewer for this suggestion. We would like to clarify that this manuscript reports on a Patient and Public Involvement and Engagement (PPIE) exercise rather than a research study. As such, the aim of the exercise was not to test a hypothesis or produce novel research data, but rather to gather patient and public perspectives to inform research design. To address the reviewer's comment, we have updated the Discussion section to reflect on findings in broader literature.*

**"Comments to the Authors **I would like to congratulate the authors on addressing an important and clinically relevant topic. The manuscript investigates an area of growing interest for GDM and the findings may have practical implications. However, there are several issues that should be addressed to strengthen the scientific quality of the manuscript."

*We thank the reviewer for their supportive comments. The manuscript has been updated in line with the recommendations as follows:*

- *The ‘Methods’ section has been revised to clarify that this was a PPIE activity, not research requiring formal qualitative or quantitative methodology. *
- *A ‘Key Findings’ title has been added at the end of the Methods section. *
- *The ‘Discussion’ section has been updated to include wider reference to existing literature. *

*We hope that this addresses the reviewer's recommendations satisfactorily.*

**
